# Adipolin and IL-6 Serum Levels in Chronic Obstructive Pulmonary Disease

**DOI:** 10.3390/arm90050049

**Published:** 2022-09-07

**Authors:** Mohammad Reza Aslani, Mojtaba Amani, Faranak Moghadas, Hassan Ghobadi

**Affiliations:** 1 Lung Diseases Research Center, Ardabil University of Medical Sciences, Ardabil 5618953141, Iran; 2 Applied Biomedical Research Center, Mashhad University of Medical Sciences, Mashhad 9177948564, Iran; 3 Department of Biophysics, School of Medicine, Ardabil University of Medical Sciences, Ardabil 5618953141, Iran; 4 Faculty of Medicine, Ardabil University of Medical Sciences, Ardabil 5618953141, Iran

**Keywords:** adipolin, IL-6, COPD

## Abstract

**Highlights:**

**What are the main findings?**

- This study showed reduced adipolin serum levels as a pro-inflammatory molecule in COPD patients.
- Serum adipolin levels correlate with serum levels of IL-6, exercise capacity (based on mMRC dyspnea score), and quality of life (based on CAT score).

**What is the implication of the main finding?**

- Imbalance in the serum levels of pro-inflammatory and anti-inflammatory markers in COPD patients.
- Correlation between pro-inflammatory and anti-inflammatory markers with disease severity and quality of life in COPD patients.

**Abstract:**

Objective(s): One of the adipokines that have insulin-sensitizing properties is adipolin, whose reduced levels have been reported in obesity, oxidative stress, and inflammation. The present study investigated serum interleukin-6 (IL-6) and adipolin levels in chronic obstructive pulmonary disease (COPD) patients. Method: A control case study included 60 COPD patients and 30 healthy subjects in the research and measured adipolin and IL-6 serum levels. In addition, serum adipolin levels in COPD patients were assessed according to the GOLD grade. The relationship between serum adipolin levels and study variables were also analyzed. Results: The results showed reduced adipolin levels in COPD patients compared with healthy individuals (*p* < 0.001). Furthermore, increased levels of IL-6 were evident in the COPD group compared to the control group (*p* < 0.001). Adipolin serum levels were positively correlated with PFTs and negatively correlated with IL-6 levels. Conclusion: Decreased adipolin levels enhanced disease severity in COPD patients. It seems that the existence of a significant relationship between adipolin and IL-6 may indicate the role of adipolin in the pathophysiology of COPD.

## 1. Introduction

Chronic obstructive pulmonary disease (COPD) is one of the significant global health risks, significantly increasing worldwide [1]. Chronic inflammation of lung airways and parenchyma is a major feature of COPD [2,3]. Although lung inflammation in patients with COPD has been extensively studied, it is not yet clear whether inflammation is associated with disease progression, response to various therapies, and/or clinical outcomes [4]. In COPD patients, systemic inflammation occurs in addition to severe airway inflammation [4]. Various inflammatory markers and cytokines play a key role in systemic inflammation, such as C-reactive protein (CRP), fibrinogen, interleukin (IL)-8, IL-6, α_1_-antitrypsin, erythrocyte sedimentation rate, and myeloperoxidase [5]. The role of adipokines secreted from adipose tissue in the pathophysiology of asthma and COPD has also been reported [6,7,8]. 

Adipokines are signaling molecules with anti-inflammatory and pro-inflammatory effects that act in an endocrine and paracrine manner [9]. Various studies have reported that adipokines can mediate many physiological changes through interactions with adipose tissue, muscles, the nervous system, and immune cells [10]. There is extensive evidence that various adipokines, such as resistin, adiponectin, visfatin, leptin, and fatty acid-binding-protein-4, participate in chronic inflammatory diseases, namely asthma, cardiovascular diseases (CVD), and COPD [11,12,13]. Adipolin (CTRP12) is highly expressed in adipocytes [14]. Adipolin has been demonstrated as an insulin-sensitizing adipokine with decreased serum levels in inflammatory disorders [15]. In obese mice, adipolin systemic administration improved glucose tolerance, proinflammatory gene expression, insulin sensitivity, and macrophage infiltration [15]. In vitro studies have also shown the inhibitory effects of adipolin on the expression of proinflammatory genes (such as TNF-α, IL-1β, and monocyte chemoattractant protein-1 (MCP-1)) [16]. Furthermore, recent evidence has demonstrated that serum adipolin levels decrease in type 2 diabetes, CVDs, and polycystic ovary syndrome (PCOS) disorders [16,17,18]. In addition, the administration of metformin significantly increases serum adipolin levels in PCOS [19]. Babapour et al. reported that in coronary artery disease (CAD) patients, there was a significant negative relationship between adipolin and troponin-T, the Gensini score, epicardial fat thickness, CK-MB, and some echocardiographic findings [17]. 

As mentioned, adipokines have been shown to play an important role in chronic diseases, such as COPD. However, the role of adipolin as an adipokine in COPD patients is not clear. Additionally, considering that there is a significant relationship between adipolin levels and some inflammatory markers, its relationship with IL-6 is not known. Therefore, the aim of the present study was to evaluate the serum level of adipolin in patients with COPD in the stable and acute exacerbation phases, as well as in healthy subjects. This study also investigated the association between serum levels of adipolin and lung function test findings and health status using the COPD assessment test (CAT score).

## 2. Methods

In this prospective cohort study, 90 male individuals were selected and divided into 30 healthy individuals (control) and 60 patients diagnosed with COPD. The sample size was selected based on the results of a previous study [17]. Exclusion criteria for the patients were surgery, pulmonary disorders other than COPD, autoimmune disorders, cancer, chronic renal failure, diabetes, cardiac ischemia, and infectious diseases. 

COPD patients were enrolled in the Department of Respiratory Medicine, and other clinics referred healthy individuals with normal spirometry. COPD diagnosis was consistent with the Global Initiative for Chronic Obstructive Lung Disease (GOLD) criteria [20]. All of the patients received their medications based on GOLD criteria and no other extra medications, such as oral corticosteroid, were prescribed. 

Pulmonary function and biochemical tests were performed for control and COPD groups on the same day. Disease severity in COPD patients was determined using GOLD grade. Applied CAT scores were used to evaluate the quality of life and the modified Medical Research Council (mMRC) to assess shortness of breath [21]. An amount of 3 to 5 cc was taken in blood samples from all participants to measure IL-6 and adipolin serum levels. After separating the serums, they were kept in microtubes at −70 °C until analyzed. Used ELISA kits (Crystal Day, Hong Kong, China) to determine biochemical parameters (IL-6 and adipolin). The absorption was determined with the microplate absorbance reader at 450 nm. The lower detection limit was 2 pg/mL for adipolin (coefficients of variation; intra-assay: 10%, inter-assay: 12%).

### Statistical Analysis

Data are reported as mean ± standard deviation (SD). A *t*-test was used to determine the differences between two groups. Correlation coefficients were assessed using the Spearman rank order test. Multivariate regression analyses were performed using adipolin as the dependent variable and IL-6, FEV1, smoking history (pack/year), and SpO_2_ as independent variables. Statistical significance was considered at *p* < 0.05. Used SPSS 21 for statistical analyses.

## 3. Results

Mean body mass index (BMI) and age were not significantly different between COPD and control groups. The pulmonary function test analysis showed that the rates of FEV1, FVC, and the FEV1/FVC ratio in the COPD patients were significantly lower than in the healthy subjects (*p* < 0.001 for all) (Table 1). 

Adipolin serum levels in the healthy group (8.88 ± 5.59) were significantly higher than in the COPD group (5.28 ± 1.28, *p* < 0.001) (Table 1). Moreover, the differences remained significant after adjusting the study groups’ adipolin results for BMI, age, and smoking history (Table 1). Elevated levels of IL-6 were also significantly seen in COPD patients (*p* < 0.001). 

**Figure 1:**
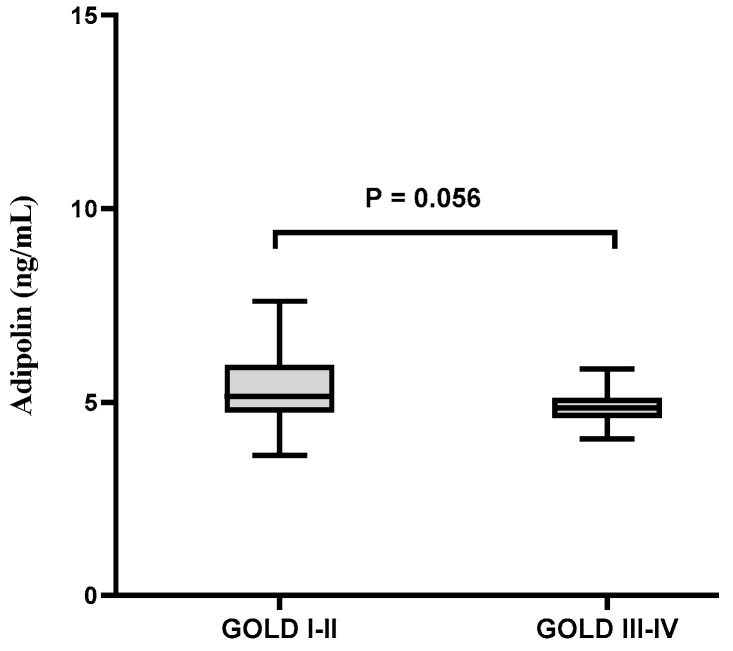
Serum levels of adipolin based on GOLD grade.

GOLD grade results identified that marked difference between stages I-II and III-IV in relation to smoking history (*p* < 0.001), SpO_2_ (*p* < 0.001), mMRC (*p* < 0.001), FEV1 (*p* < 0.001), CAT score (*p* < 0.001), and IL-6 (*p* < 0.01, Table 2). Based on the GOLD grade, it was revealed that adipolin levels in GOLD III-IV were marginally significant compared to GOLD I-II. (*p* = 0.056, Figure 1).

### Relationship of Serum Levels of Adipolin and IL-6 with the Study Parameters

**Figure 2:**
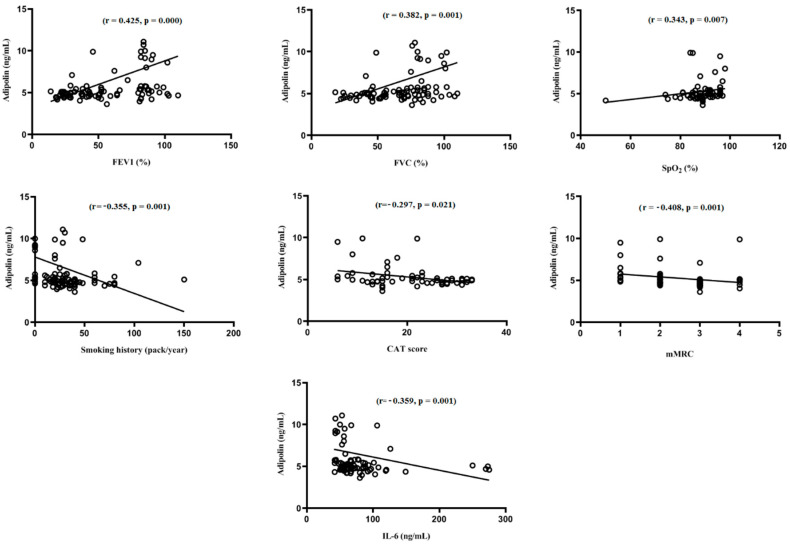
Spearman correlation analysis of study parameters with adipolin. FEV1: forced expiratory volume in 1 s, FVC: forced volume capacity, IL-6: interleukin 6, SpO_2_: O_2_ saturation, GOLD: the Global Initiative for Chronic Obstructive Lung Disease, mMRC: modified medical research council, CAT: COPD Assessment Test.

Parameters that had a significant positive correlation with serum adipolin levels were FEV1_% predicted_ (*p* < 0.001), FVC (*p* < 0.001), FEV1/FVC ratio (*p* < 0.01), and SpO_2_ (*p* < 0.01). On the other hand, serum adipolin levels were negatively correlated with smoking history, IL-6, mMRC, and CAT score (Figure 2). Furthermore, significant relationships between IL-6 levels and FEV1 (r = −0.634, *p* < 0.001), FVC (r = −0.534, *p* < 0.001), FEV1/FVC (r = −547, *p* < 0.001), SpO_2_ (r = −0.620, *p* < 0.001), mMRC (r = 0.629, *p* < 0.001), smoking history (r = 0.547, *p* < 0.001), and CAT score (r = 0.419, *p* < 0.01) were observed.

Multiple regression tests revealed that among the variables, smoking history (pack/year), FEV1, IL-6, and CAT score, only FEV1_% predicted_ (*p* < 0.05) was used as a predictor of adipolin levels (Table 3).

## 4. Discussion

The results showed significantly reduced adipolin serum levels in COPD patients compared with healthy subjects for the first time. It was also found that the serum level of adipolin was significantly associated with parameters such as FEV1, smoking history, mMRC, CAT score, and IL-6. Moreover, multiple regression analysis revealed that only FEV1 was independently predicted from adipolin serum levels. 

In human and animal studies, it has been identified that in inflammatory disorders, such as diabetes, CVDs, non-alcoholic fatty liver, and metabolic syndrome, there is a disruption in serum and gene expression levels of CTRP family members [17,22,23,24]. Adipolin (CRTP12) is an adipocytokine with insulin-sensitizing properties, primarily expressed in adipose tissue [25]. In patients with COPD and asthma, it has been shown that adipokines play a crucial role in generating chronic low-grade inflammation [2]. Decreased adipolin levels have been reported in CVDs, PCOS, and type 2 diabetes [17]. For the first time, the current study results demonstrated reduction in adipolin serum levels in the COPD group compared with healthy individuals. Furthermore, the results showed a significant negative correlation between adipolin and IL-6 levels. 

It has been determined that types of inflammatory cells, namely macrophages, play a crucial role in COPD pathophysiology [2]. Macrophages are an important source of various cytokines, i.e., TNF-α and IL-6, which, by activating macrophages, induce adipokines secretion [2]. Enomoto et al. experimentally demonstrated that the upregulation of adipolin mediated by adenovirus led to reduced recruitment of macrophages and suppressed expression levels of MCP-1, TNF-α, and IL-1β [25]. They also reported that the upregulation of MCP-1, IL-1β, and TNF-α induced by lipopolysaccharides (LPS) was significantly reduced by adipolin [25]. In addition, Tan et al. reported a significant negative association between adipolin and C-reactive protein [19]. Furthermore, Fadaei et al., in patients with coronary artery diseases, identified a negative relationship between adipolin levels and proinflammatory markers such as TNF-α and IL-6 [18]. The present study results also revealed a negative association between adipolin and IL-6 in COPD patients. Considering that in animal studies [25] a decreased level of adipolin has been reported in an inflammatory environment, it is possible, at least in part, to infer that the presence of chronic inflammatory conditions in COPD patients affects the reduced serum levels of adipolin, which should be further investigated. 

Decreased adipolin serum levels in the current study were also associated with the severity of the diseases. It was revealed that adipolin levels in GOLD III-IV were marginally significant compared to GOLD I-II. Perhaps the lack of significance was due to the small sample size, which requires additional studies. According to the study results, the negative relationship between adipolin and IL-6 may indicate the existence of a complex regulatory system that affects the severity of the disease. 

Although the exact pathophysiological mechanism of adipolin in COPD patients is not clear, the anti-inflammatory effects of adiploin can be deduced from previous studies. Animal and human studies have demonstrated that adiponectin levels (part of CTRP family) are negatively correlated with disease severity of COPD and asthma patients [26]. Most findings indicate that the role of adiponectin in inflammatory processes is inhibition of proinflammatory markers (such as VCAM-1, ICAM-1, IL-6, NF-kB, and TNF-α,) or promotion of anti-inflammatory mediators (such as IL-10) [27,28]. Some contradictory results have been reported about serum levels of adiponectin in human studies. Therefore, for a solid understanding, further investigations are needed to justify the role of the CTRP family in COPD patients [26,29].

A significant negative correlation between adipolin and health assessment criteria and the mMRC scale in COPD group was also revealed. The activity level of patients with COPD is significantly reduced, which is more pronounced in the late stages of the disease [30]. Although the primary mechanism of the decrease of activity in COPD patients is unknown, an increase in IL-6, CRP, and fibrinogen has been implicated in this mechanism [31]. Decreased adipolin and elevated IL-6 levels in the upper GOLD grade of the disease may indicate the role of systemic inflammation in the health status of COPD patients.

The limitations of this study can be summarized as follows: we did not look into the impact of gender on serum adipolin levels. High serum adipolin levels have been reported in women compared to men [18]. Moreover, the protective effects of adipolin have been demonstrated in various studies against inflammatory markers. Evaluating the impact of adipolin on inflammatory responses in COPD patients seems useful. A large sample size study could also be significant in more accurately assessing serum adipolin levels in COPD patients.

## 5. Conclusions

The current study showed the reduced adipolin serum levels in COPD patients for the first time. Moreover, the results indicated a correlation between adipolin and FEV1, IL-6, mMRC, SpO_2_, and CAT scores. Decreased adipolin levels, and their correlation with IL-6, appear to have contributed to the pathogenesis of the disease through changes in inflammatory conditions, which requires further study. 

## Figures and Tables

**Table 1:** Baseline characteristics of patients with COPD and control subjects.

Parameters	Control Group (*n* = 30)	COPD Group (*n* = 60)	*p*-Value
Mean age (year)	56.40 ± 6.29	58.92 ± 6.22	0.099
Body mass index (kg/m^2^)	26.73 ± 3.88	24.72 ± 4.79	0.051
Smoking history (pack/year)	13.03 ± 13.61	38.83 ± 25.34	0.000
Pulmonary function test:			
FEV1 (% of predicted)	89.06 ± 7.60	43.82 ± 20.59	0.000
FVC (% of predicted)	85.50 ± 8.79	58.71 ± 22.42	0.000
FEV1/FVC	83.65 ± 5.89	59.43 ± 11.69	0.000
Adipolin (ng/mL)	8.88 ± 5.59	5.28 ± 1.28	0.001
Adjusted adipolin	8.89 ± 0.34	5.28 ± 0.61	0.000
IL-6 (ng/mL)	54.56 ± 10.47	87.71 ± 52.92	0.001
Adjusted IL-6	52.22 ± 14.09	88.41 ± 30.86	0.000

Data are presented as mean ± SD. FEV1: forced expiratory volume in 1 s, FVC: forced volume capacity, IL-6: interleukin 6.

**Table 2:** GOLD groups and baseline characteristics of the study population.

Variables	GOLD I-II	GOLD III-IV	*p*-Value
Number	22	38	
Age (year)	58.41 ± 6.55	59.21 ± 6.10	0.642
BMI (kg/m^2^)	24.53 ± 3.84	24.83 ± 5.31	0.817
Smoking (pack per year)	21 (20–40)	38 (28–60)	0.008
CAT score	13 (9–15)	27 (22–29)	0.000
mMRC	2 (1–2)	3 (2–3)	0.000
IL-6 (ng/mL)	62.81 ± 10.56	102.13 ± 61.82	0.005
Adipolin (ng/mL)	5.69 ± 1.63	5.04 ± 0.96	0.056

Data are depicted as mean ± SD or median (25–75th percentiles). GOLD: the Global Initiative for Chronic Obstructive Lung Disease, COPD: chronic obstructive pulmonary disease, BMI: body mass index, IL-6: interleukin-6, CAT: COPD Assessment Test, mMRC: modified medical research council.

**Table 3:** Multivariate analysis between adipolin and study parameters.

Adipolin			
	B	95% CI for B	*p*-Value
FEV1	0.544	0.009–0.058	0.008
cigarette history (pack/year)	0.117	−0.010–0.022	0.459
IL-6	0.045	−0.007–0.009	0.781
CAT score	0.031	−0.056–0.066	0.873

B represents the unstandardized coefficient. CI: confidence intervals, FEV1: forced expiratory volume in 1 s, IL-6: interleukin-6.

## Data Availability

All data are available whenever needed through the corresponding author.
